# Development and validation of the Healthy-Unhealthy Music Scale

**DOI:** 10.1111/camh.12109

**Published:** 2015-05-18

**Authors:** Suvi Saarikallio, Christian Gold, Katrina McFerran

**Affiliations:** 1 Department of Music, University of JyväskyläPoBox 35, Jyväskylä, FI-40014, Finland; 2 Uni Research, GAMUTBergen, Norway; 3 Melbourne Research, The University of MelbourneMelbourne, Victoria, Australia

**Keywords:** Music use, adolescents, depression, prevention, mental health, scale development

## Abstract

**Background:**

Music is an integral part of life in youth, and although it has been acknowledged that musical behavior reflects broader psychosocial aspects of adolescent behavior, no measurement instruments have been specifically designed for assessing musical engagement as an indicator of adolescent wellbeing and/or symptomatology. This study was conducted in order to develop and validate a scale for assessing musical engagement as an indicator of proneness for depression in youth.

**Method:**

Items were developed based on the literature and a prior grounded theory analysis and three surveys (*N* = 54, *N* = 187, *N* = 211) were conducted to select, refine, test, and validate the items. Scale structure was investigated through interitem correlations, exploratory and confirmatory factor analyses (EFA, CFA), and concurrent validity was tested with correlations to depression and wellbeing.

**Results:**

The final Healthy-Unhealthy Music Scale (HUMS) consists of 13 items that are divided into Healthy and Unhealthy subscales. Cronbach's alpha coefficients were .78 for Healthy and .83 for Unhealthy. The concurrent validity of the HUMS was confirmed through correlations to wellbeing, happiness and school satisfaction on one hand and depression, rumination, and stress on the other.

**Conclusions:**

The HUMS is as a promising instrument for screening musical engagement that is indicative of proneness for depression in youth.

**Key Practitioner Message**

- No prior scales directly measuring music as an indicator of wellbeing and/or symptomatology in youth
- Healthy-Unhealthy Music Scale (HUMS) was developed to measure music engagement from the perspective of proneness for adolescent depression
- HUMS was established as a reliable, valid, easy to use, brief measure for assessing musical engagement as an indicator of proneness for depression in youth

## Introduction

Research into the relationship between young people, music, and mental health is proliferating in the face of the increasing access to daily music ‘doses’ enabled by the digital revolution. This coincides with a growing interest in how relevant music is to health (McDonald, Kreutz, & Mithcell, 2012), to emotion (Juslin & Sloboda, 2010), and to identity (McDonald, Hargreaves, & Miell, 2002). Policy statements about the impact of music also surface (Fuld et al., 2009). Nevertheless, the pathways that connect music to mental health in youth are poorly understood and we lack reliable assessment of young people's musical engagement from the perspective of health-relevance.

There is mounting evidence of the health benefits of music. Music-based interventions efficiently reduce stress and improve mood (Clark & Harding, 2012; Erkkilä, Punkanen, Fachner, & Gold, 2011; Field et al., 1998; Maratos, Gold, Wang, & Crawford, 2008; Menon & Levitin, 2005; Pelletier, 2004) and it has been argued that music can support adolescent development regarding identity, interpersonal relationships, self-agency, coping, and mood regulation (Gold, Saarikallio, & McFerran, 2011). In contrast, however, music engagement also relates to measures of ill-health including internalized symptomatology and depression (Doak, 2003; Lacourse, Claes, & Villeneuve, 2001; McFerran, Garrido, & Saarikallio, 2013; Miranda & Claes, 2009; Miranda, Gaudrea, Debrosse, Morizot, & Kirmayer, 2012) as well as externalized symptoms and antisocial behaviors (Mulder, Ter Bogt, Raaijmakers, & Vollebergh, 2007; North & Hargreaves, 2012).

What kind of musical engagement, then, is indicative of wellbeing and when does music reflect mental health problems? The type of musical activity may be of relevance: listening to music with violent lyrics increased hostility (Anderson, Carnagey, & Eubanks, 2003), while active participation in drumming decreased aggression (Currie & Startup, 2012). Attachment to certain ‘problem’ genres might also indicate vulnerability to mental health problems (North & Hargreaves, 2012). However, this ‘problem’ music may actually serve positive functions in the lives of the young (Lozon & Bensimon, 2014).

Indeed, the health-relevance of music cannot be defined by a single musical act or a particular genre preference, but needs to be considered within the broader context of the individual (McFerran & Saarikallio, 2013). A particular musical behavior (e.g. listening to sad music that reflects one's sadness) may be favorable in one context, or for a period of time (grieving in a funeral, dealing with particular situation), but may, if prolonged, and coupled with vulnerability for depression, become maladaptive (a cycle of self-pity, rumination, and depression). This way, music interacts with the context of each young person and their existing state of mental health to potentially intensify experiences and behavioral patterns (McFerran & Saarikallio, 2013). At-risk individuals may couple music with their maladaptive behavioral patterns and, therefore, their uses of music can provide clues toward the assessment of their mental health (Brown & Hendee, 1989).

What then, are the behavioral patterns of musical engagement that reflect vulnerability of depression in youth? A variety of music-related mood regulation strategies have been identified (Saarikallio, 2008; Saarikallio & Erkkilä, 2007; ), and these strategies appear to mediate the health-effects of musical engagement (Miranda & Claes, 2009; Miranda et al., 2012). Depression has been shown to relate to tendencies for employing music for avoidant coping (Miranda & Claes, 2009) and rumination (Garrido & Schubert, 2013). Garrido and Schubert (2013) argue that musical engagement of depressed individuals is indicative of the maladaptive regulation strategies that underpin their diagnosis, including a tendency to justify the ‘bittersweet’, nonmood-improving, result of listening. In contrast, music listening relates to positive health-outcomes particularly if combined with mood enhancement, reappraisal, and distraction (Chin & Rickard, 2014; Edwards, 2011; Van den Tol & Edwards, 2013, 2014).

As a preparation for this study, we further consolidated the theoretical understanding of the phenomenon through a qualitative study with depressed and nondepressed adolescents (McFerran & Saarikallio, 2013). The results showed that the musical engagement of the depressed adolescents was characterized by ruminative thinking through repetitive listening, by escape and avoidance of problems and troubled relationships through music listening, and by a tendency of music listening to result in mood worsening toward sadness, anger, negative energy, and frustration. Furthermore, depressed adolescents were reluctant to acknowledge the ‘failed attempts’ and situations when music did not lead to desired healthy outcomes: they appeared unwilling to change their listening behavior accordingly and typically reflected on these experiences only retrospectively when moving toward recovery. The results were consistent with previous research about how musical engagement relates to depression versus positive health-indicators also in adult population (Chin & Rickard, 2014; Edwards, 2011; Garrido & Schubert, 2013; Van den Tol & Edwards, 2013, 2014).

This paper reports the development and validation of a measurement scale for assessing the music engagement of young people as an indicator of healthy/unhealthy behavior, from the particular perspective of proneness for depression. We describe how items were developed, how they were tested, refined, and selected, and how scale reliability and validity were established.

## Methods

### Participants

Three surveys with young people (13–20 years) were conducted with two samples of younger (13–15 and 13–17 years) and one sample of older adolescents (19–20 years). This variability was considered acceptable as previous research indicates no major differences between adolescents and adults in how patterns of musical behavior link to depression (McFerran & Saarikallio, 2013). Informed consent for all surveys and ethical statements for studies with underaged participants were obtained (Ethics ID # 1034456.1 & # 1137267). While young people clinically diagnosed with depression participated in piloting the items, the two latter surveys were conducted with normal populations with self-report measures of depression. This enabled wide samples necessary for statistical testing and was considered appropriate since HUMS was aimed for preventive screening purposes in general population.

### Item development

The first set of items for the Healthy-Unhealthy Music Scale (HUMS) was formulated based on previous literature and designed to reflect such features of musical engagement that relate either positively or negatively to depression. The item content concerned moods, emotions, coping, interpersonal relationships, self-esteem, and identity. As previous research indicates that it is not so much the type of musical act per se (using music to match one's mood) but rather the outcome (worsening of mood) that relates to depression, the outcomes of musical engagement were emphasized in the items. Response categories were on a 5-point scale from ‘never’ to ‘always’. Item wordings were discussed internally and with colleagues from the field.

### Piloting the items

Healthy-Unhealthy Music Scale version 1 (with 21 items) was piloted with 39 nondepressed adolescents (13–14 years) and 15 adolescents (13–17 years) clinically diagnosed with depression and receiving inpatient psychiatric treatment for depression. Qualitative comments from adolescents and their teachers were collected about the item wording and statistical analyses were conducted to identify which items would best differentiate between the samples (comparison of means, *t*-tests) and result in a clear internal structure (exploratory factor analysis, EFA). We aimed for items having a mean difference between the samples significant and larger than half a point in the scale and items having factor loadings > .50 for the respective factor and cross-loadings <.30 on other factors. Based on statistical results and qualitative feedback, HUMS was revised to version 2 with 36 items.

### Selecting the items

Healthy-Unhealthy Music Scale version 2 was completed by young University students (*N* = 187, aged 19–20) along with a question about how often they had felt depressed during the last month, answered with a five-point scale ranging from *none of the time* to *all of the time* (modified from the Kessler Psychological Distress Scale, (K10) (Kessler et al., 2002).

We sorted the HUMS 2 items by their correlation with the depression score. Using Cohen's (Cohen, 1988) guidelines for small (*r* = .10) and medium (*r* = .30) effect sizes, we retained items correlating with depression by ≥ .10 in the expected direction. Duplicate items (conceptually repeating another item) were removed if another item on the topic had a higher correlation (in the expected direction). Selected items were subjected to investigation of means and standard deviations, correlations between each other, and exploration of the scale structure through EFA (using Maximum Likelihood for extraction and Promax for rotation as factors were expected to correlate).

### Confirming scale structure and testing reliability

In a third survey 211 adolescents (13–15 years) recruited from Australian schools completed the third, 14-item, version of HUMS. The structure of the near-final HUMS version 3 was again examined through correlations and EFA (using ML and Promax) to identify any items to be removed. Confirmatory factor analysis (CFA) was subsequently used to confirm the proposed factor structure. Measurement models for a two-factor and a three-factor solution were tested using IBM Amos software version 22.0 with maximum likelihood (ML) estimation. The model fit was tested with *χ*^2^-test (nonsignificant values indicate a good model), comparative fit index (CFI; values should be greater than .90, preferably greater than .95 to consider a good fit; Hu & Bentler, 1999), root-mean square error of approximation (RMSEA; values .05 or below indicate a good model; Browne & Cudeck, 1993), and the root-mean square residual (RMR; values .05 or below indicate a good model; Jöreskog & Sörbom, 1996). Having thus established the final version with two subscales (HUMS Healthy & HUMS Unhealthy), we computed reliability coefficients for both subscales.

### Testing validity

Survey 3 contained measures of depression and mental wellbeing to assess the concurrent validity of HUMS. Depression was measured through the K10, a global measure of distress consisting of 10 questions about anxiety and depressive symptoms experienced during the last month and mental wellbeing through the Mental Health Continuum Short Form (MHC-SF) (Keyes, 2002) that consists of three subscales (psychological, emotional, and social wellbeing) summed into a total score. Both K10 and MHC-SF have been validated for adolescents and have shown excellent psychometric properties in previous studies (Andrews & Slade, 2001; Kessler et al., 2002; Keyes, 2002, 2005; Lamers, Westerhof, Bohlmeijer, ten Klooster, & Keyes, 2010). Three additional questions were included to investigate happiness, school satisfaction, and self-perceived stress: ‘I generally feel happy’; ‘School days give me a feeling of accomplishment’; ‘Do you feel that kind of stress at the moment?’ (combined with a description of stress consisting of both long-term and short-term stressors and related experiences). Finally, as HUMS covers musical rumination the Rumination-Reflection Questionnaire (RRQ) (Trapnell & Campbell, 1999) was included. The scale has a clear two-factor structure and consists of two 12–item subscales: the rumination subscale relates to neuroticism, depression and negative affect, while the reflection subscale mostly relates to the personality trait of openness (Trapnell & Campbell, 1999). An example of rumination item is ‘Long after an argument or disagreement is over with, my thoughts keep going back to what happened’. All measures were answered with 5-point Likert scales.

Healthy-Unhealthy Music Scale Healthy was expected to correlate positively and HUMS Unhealthy negatively with indicators of good mental health, including wellbeing (MHC-SF), happiness, school satisfaction, and, possibly, reflection, health connections of which have only been tentative so far (Trapnell & Campbell, 1999). Conversely, HUMS Healthy was expected to correlate negatively and HUMS Unhealthy positively with rumination, self-perceived stress, and, in particular, depression (K10).

## Results

### Item characteristics of HUMS version 1

For both samples, most item means were between 2 and 4, and *SD*s around 1, indicating an adequate spread. Differences between the samples greater than 0.5 in the expected direction were only found for three items; many of the differences were in the expected direction but smaller. EFA suggested a four-factor model (First four factors with eigenvalues over 1.0 explained 74.7% of total variance), in which all ‘healthy’ items loaded on factors 1 and 2 and most ‘unhealthy’ items on factors 3 and 4, showing that the items grouped according to the healthy-unhealthy dimension. Thus, most items showed ‘right direction’ regarding the health-connection, but not strongly or clearly enough (also having a cross-loading and/or showing relatively low mean difference between the samples). This lead us to discuss the possible reason for such a result regarding each item, rephrase some items accordingly, and augment set to 36 items with some duplicate/optional items for identifying the best wording for each item in survey 2.

### Item selection through survey 2

Mean score for the self-rated depression in the survey 2 sample was 2.57 (*SD* = .81, range 1–5). HUMS version 2 items and their correlations with depression are shown in Table 1. Based on the criteria presented in Methods, we retained nine ‘unhealthy’ and five ‘healthy’ items (shaded gray). Reasons for removal are listed in Table 1.

**Table 1:** Healthy-Unhealthy Music Scale (HUMS) version 2 items with their correlation with depression, underlying concept, and reason for removal, with HUMS version 3 items presented with means, standard deviations, and factor loadings for their respective factor

Items	*r*	Concept	Reasons for removal	*M* (*SD*)	Factor (loading)
When I try to use music to feel better I actually end up feeling worse	.31	M		1.91 (.81)	F1 (.40)
I hide in my music because nobody understands me, and it blocks people out	.31	S		2.04 (1.10)	F2 (.55)
Music gives me an excuse not to face up to the real world	.31	S		1.97 (.95)	F2 (.86)
I use music to escape really hard feelings	.28	M	Loaded on healthy factor in pilot phase		
I relate more to my favourite lyrics than to what my friends have to say	.25	S		2.22 (.95)	F1 (.28) F2 (.26)
When I listen to music I get stuck in bad memories	.24	M		2.16 (.89)	F1 (.84)
I like to listen to songs over and over even though it makes me feel worse	.23	M		2.47 (1.16)	F1 (.72)
My music is like a friend who gets me	.22	S	Loaded on healthy factor in pilot phase		
It can be hard to stop listening to music that connects me to bad memories	.20	M		2.74 (1.16)	F1 (.73)
When I feel bad I listen to the same song over and over again	.17	M	Items with higher correlation on this concept		
After engaging with music I feel stronger	.16	M	Expected to be healthy		
Music makes me feel bad about who I am	.14	I		1.38 (.65)	F2 (.49)
Music leads me to do things I shouldn't do	.14	C		1.67 (.84)	F2 (.38)
If I'm in a bad mood and then I use music to express myself I feel better	.14	M	Expected to be healthy		
I use music when I don't want to talk to other people	.12	S	Items with higher correlation on this concept, Loaded on healthy factor in pilot phase		
Music can pump me up to do things that could be described as unhealthy	.12	C	Items with higher correlation on this concept		
I'm afraid to share my music with other people in case they don't appreciate it	.10	S	No correlation		
Listening to the lyrics of songs helps me to sort out my problems	.09	C	No correlation		
After hearing my music people know how I feel	.06	S	No correlation		
I feel bad when I compare myself to my favorite musicians	.05	S	No correlation		
I can tell other people how I feel through music	.04	S	No correlation		
I'm afraid to share my music with others	.04	S	No correlation		
I spend so much time on music that I have no time for study and other stuff I should do	.04	F	No correlation		
I feel proud of myself after I share my music with others	.02	S	No correlation		
Sharing music with my friends makes me feel part of the group	.02	S	No correlation		
Music helps me to express who I am	.00	I	No correlation		
When I'm really stressed music makes me feel light again	−.03	M	No correlation		
Listening to music helps me to get my jobs done	−.04	C	No correlation		
My music brings me pleasure	−.07	M	No correlation		
I get my jobs done faster when I'm listening to music	−.09	F	No correlation		
When I'm in a bad mood music helps me to feel better	−.10	M	No correlation		
Music helps me to connect with other people who are like me	−.13	S		3.41 (1.02)	F3 (.38)
Music helps me to relax	−.16	C		4.10 (.76)	F3 (.62)
Music gives me the energy to get going	−.17	M,C		3.69 (.80)	F3 (.54)
When I'm feeling tense or tired in my body music helps me to relax	−.17	C		3.85 (.74)	F3 (.64)
I feel happier after playing or listening to music	−.18	M		3.97 (.62)	F3 (.52)

M, Mood; C, Coping; S, Social/Interpersonal; F, Focus/Self-efficacy; I, Identity; F1, Factor1; F2, Factor2; F3, Factor3. Items are ordered according to their correlation (*r*) with depression, and the reason for removal is provided for items that were not retained. The 14 items retained for the HUMS version 3 are shaded with gray and information is provided regarding their means and standard deviations and the factor loadings for their respective factor in the EFA.

Means and *SD*s (Table 1) suggested possible floor effects for two unhealthy items. These were subsequently modified by adding the verb ‘can’: ‘When I try to use music to feel better I *can* actually end up feeling worse’ and ‘When I listen to music I *can* get stuck in bad memories’.

Among the 14 items thus retained, we found positive correlations between unhealthy items (ranging from .10 to .62) as well as between healthy items (.17 to .48). Correlations between healthy and unhealthy items were typically around zero (ranging from −.29 to .14). EFA indicated a 3-factor structure: The first three factors had eigenvalues above 1.0 and explained 50.3% of the variance. Factor loadings (Table 1) now indicated a clear division between healthy and unhealthy factors. All unhealthy items loaded on Factors 1 (rumination) and 2 (avoidance and social alienation) and all healthy items on Factor 3. These analyses suggested that the 14 items selected for HUMS version 3 were applicable in reflecting one or the other end of the healthy-unhealthy dimension of music use.

### The final HUMS scale: structure and reliability

Healthy-Unhealthy Music Scale version 3 was applied in survey 3. Again, correlations between healthy items (ranging from .36 to .53) and unhealthy items (.05 to .59) were positive, and those between healthy and unhealthy items were mostly low (−.19 to .28). Item ‘I relate more to my favourite lyrics than what my friends have to say’ was ambiguous, correlating positively with unhealthy (.05 to .49) and healthy items alike (.18 to .35). EFA indicated a two-factor solution (first two factors with eigenvalues above 1.0 explained 50.0% of the variance) with Factor 1 reflecting unhealthy and Factor 2 healthy dimensions. All items, except two, had loadings over .50 on their respective factor with no cross-loadings. ‘Music leads me to do things I shouldn't do’ had a loading of only .44 on its respective factor, but no cross-loadings. Due to a relatively high loading and clear health-direction this item was retained. Meanwhile, ‘I relate more to my favourite lyrics than what my friends have to say’, loaded similarly on both factors. We removed this item from the final scale and reran the EFA with the remaining 13 items. Results were almost identical, again suggesting a two-factor solution (two factors with eigenvalues >1, explaining 50.8% of the variance; Factor 1 representative of unhealthy and Factor 2 of healthy dimension). The factor loadings (pattern matrix) are presented in Table 2.

**Table 2:** The factor loadings (pattern matrix) of the final version of Healthy-Unhealthy Music Scale

Items	F1	F2
When I listen to music I get stuck in bad memories	**.760**	−.033
I like to listen to songs over and over even though it makes me feel worse	**.714**	−.092
It can be hard to stop listening to music that connects me to bad memories	**.658**	.187
I hide in my music because nobody understands me, and it blocks people out	**.639**	.156
When I try to use music to feel better I actually end up feeling worse	**.627**	−.163
Music gives me an excuse not to face up to the real world	**.571**	.249
Music makes me feel bad about who I am	**.521**	−.186
Music leads me to do things I shouldn't do	**.428**	−.103
I feel happier after playing or listening to music	−.157	**.708**
Music gives me the energy to get going	−.005	**.692**
When I'm feeling tense or tired in my body music helps me to relax	−.028	**.667**
Music helps me to relax	.040	**.621**
Music helps me to connect with other people who are like me	−.061	**.608**

Exploratory factor analysis with promax rotation. Factor loadings greater or equal 0.4 are highlighted in bold font.

**Figure 1:**
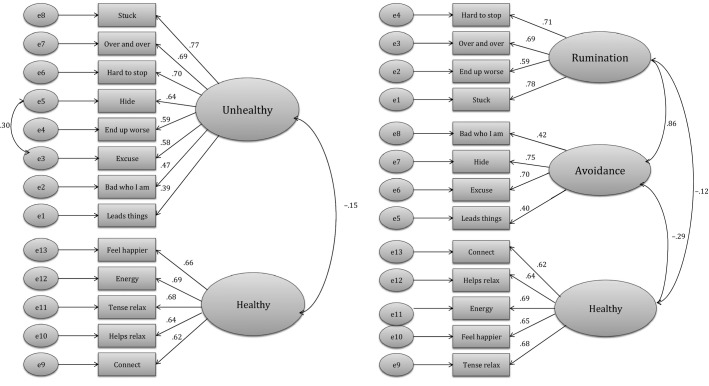
The estimated two-factor and three-factor measurement models of Healthy-Unhealthy Music Scale (standardized solutions)

Finally, since survey 2 had indicated a three-factor solution, CFA was used to confirm whether a two-factor or a three-factor solution would provide the best fit for the data. The two-factor measurement model consisted of one unhealthy and one healthy factor and the three-factor model consisted of two unhealthy factors (F1 = rumination, F2 = avoidance, based on EFA in survey 2) and one healthy factor. The measurement models of both solutions with standardized estimates are presented in Figure1. Based on modification indices, the measurement errors of two unhealthy items, measuring a similar subtopic of avoidance, were allowed to correlate with each other in the two-factor solution. The reliability coefficients for all observed items were above .30 in both models and the fit indices indicated an adequate fit for both models (two-factor model: *χ*^2^ (63) = 136.549, CFI = .908, RMSEA = .075, RMR = .088; three-factor model: *χ*^2^ (62) = 137.320, CFI = .906, RMSEA = .076, RMR = .079). The results showed that HUMS items could be grouped either to two or three factors. However, as correlation between the two unhealthy factors was as high as .86 and, theoretically, both of these factors were illustrative of the same health-dimension, the more concise two-factor solution was considered preferable. This decision was further supported by the practical viewpoint of constructing as straightforward as possible an instrument with maximum ease of scoring and use by clinicians.

The final version of HUMS was thus established as a 13-item scale, consisting of five healthy and eight unhealthy items, subsequently divided into two subscales: HUMS Healthy and HUMS Unhealthy (see Appendix S1 available online). As designed, the scale reflected the healthy-unhealthy dimension and contained but did not separate between conceptual subcomponents such as rumination and avoidance. Internal consistency of both subscales was acceptable (Cronbach's alpha: .78 for HUMS Healthy; .83 for HUMS Unhealthy).

### The concurrent validity of HUMS

Next, the concurrent validity of HUMS was assessed. Mean scores for all measures in boys and girls are presented in Table 3. The overall mean score for depression was 21.02 (*SD* = 7.77; range 10–48), falling within a moderate level of distress. Girls scored significantly higher than boys for HUMS Unhealthy, depression, rumination, and stress, while boys scored significantly higher than girls for wellbeing (Table 3). None of the measures correlated with age. Due to the observed gender differences we calculated partial correlations controlling for gender when testing connections between measures (Table 4).

**Table 3:** Means and standard deviations for all measures in boys and girls

Measure	Boys *M* (*SD*)	Girls *M* (*SD*)	*t*	*df*	*p*
HUMS Healthy	19.66 (3.35)	20.27 (3.59)	−1.25	209	.121
HUMS Unhealthy	14.45 (5.11)	17.41 (6.26)	−3.78	209	.000***
Depression (K10)	19.61 (6.76)	22.18 (8.36)	−2.45	208.97	.014*
Wellbeing (MHC-SF)	49.82 (11.91)	46.22 (14.21)	2.00	208.97	.047*
Rumination (RRQ)	3.14 (.68)	3.36 (.75)	−2.19	209	.030**
Reflection (RRQ)	3.02 (.40)	3.06 (.51)	−.74	208.78	.452
Happiness	4.04 (.78)	3.84 (.90)	1.68	208.18	.091
School satisfaction	3.29 (.98)	3.34 (.92)	−.32	209	.752
Stress	2.27 (.95)	2.72 (1.11)	−3.13	209	.002**

* *p* < .05;

** *p* < .01;

*** *p* < .001.

**Table 4:** Partial correlations (controlling for gender) for the Healthy-Unhealthy Music Scale subscales and the other measures

	HUMS Healthy	HUMS Unhealthy
HUMS Healthy	1	
HUMS Unhealthy	.14*	1
Depression (K10)	.18**	.67***
Wellbeing (MHC-SF)	.09	−.48***
Rumination (RRQ)	.21**	.50***
Reflection (RRQ)	.24***	.01
Happiness	.21**	−.38***
School satisfaction	.29***	−.19**
Stress	.08	.40***

* *p* < .05;

** *p* < .01;

*** *p* < .001.

Correlations between the measures of depression, rumination, reflection, stress, wellbeing, happiness, and school satisfaction were in the expected directions (not shown). As expected, HUMS Unhealthy correlated positively with depression (*r* = .67, *p* < .001), rumination (*r* = .50, *p* < .001), and stress (*r* = .40, *p* < .001) and negatively with wellbeing (*r* = −.48, *p* < .001), happiness (*r* = −.38, *p* < .001), and school satisfaction (*r* = −.19, *p* < .01). No correlation with reflection was found. All of these correlations were consistent with expectations, confirming the validity of HUMS Unhealthy for assessing musical engagement as an indicator of mood and mental health problems.

Correlations of HUMS Healthy were more mixed, showing significant correlations in the expected direction with reflection (*r* = .24, *p* < .001), happiness (*r* = .21, *p* < .01), and school satisfaction (*r* = .29, *p* < .001), but also low correlations in the unexpected direction with depression (*r* = .18, *p* < .05) and rumination (*r* = .21, *p* < .01) and no correlation with stress and wellbeing (Table 4). Furthermore, low positive correlation was found between the HUMS subscales (*r* = .14; *p* < .05; Table 4), indicating a shared underlying element, plausibly the engagement in *music* for socioemotional purposes.

## Discussion

A new measurement instrument labeled Healthy-Unhealthy Music Scale was developed and tested. The field of music and adolescent mental health has sorely lacked instruments to assess musical engagement from the perspective of mental health, and HUMS is a pioneering tool for this purpose. The strength of HUMS lies in solid qualitative theory development underpinning the item construction – the items are strongly rooted both in previous literature and in grounded theory analysis of music uses of depressed and nondepressed young people. The scale's focus on depression-related music use has been consistently maintained throughout the scale development process, which makes HUMS a theoretically well-grounded instrument.

The HUMS consists of 13 items, of which five measure healthy and eight unhealthy dimensions of musical engagement. It reflects several key components previously identified as relevant for health-related music use: the Healthy subscale includes social connection (McFerran & Saarikallio, 2013), mood enhancement, and distractive relaxation (Edwards, 2011; Van den Tol & Edwards, 2013, 2014) and the Unhealthy subscale contains avoidant coping (Miranda & Claes, 2009), rumination (Garrido & Schubert, 2013), and mood worsening, even despite contrasting intention (McFerran & Saarikallio, 2013). The scale was, however, not designed to separate between all these concepts. This was reflected in the scale structure: Both exploratory and confirmatory factor analyses consistently suggested a separation into healthy versus unhealthy factors, not necessarily into further factors. Thus, despite the presence of different underlying concepts imbedded in it, we recommend HUMS to be used primarily as a measure of the healthy-unhealthy dimension, rather than separating it into subelements such as avoidance and rumination.

The reliability and validity tests provided favorable and confirmative results. The internal consistency and concurrent validity of HUMS Unhealthy were excellent. It correlated strongly with depression, rumination, and wellbeing, and moderately also with stress, happiness, and school satisfaction thus presenting itself as highly reliable and valid measures of musical engagement that indicates risk or proneness for depression in youth.

Healthy-Unhealthy Music Scale healthy had equally excellent internal consistency but its concurrent validity was ambiguous. HUMS Healthy correlated in the expected direction with happiness, reflection, and school satisfaction, but not with rumination and depression: this subscale or its items correlated positively with depression in the last survey, but negatively in previous samples. This is likely to be due to the complexity of the health-promoting potential of music use. Young people prone to depression are likely to be in greater need for an extra medium (music) to support coping, and may manage to use it not only in unhealthy but also in healthy ways,. Music use increases when there is an increase of problems in youth (Arnett, 1995) and music may indeed hold particular potential to help young people to process their existing negative emotional states (Lozon & Bensimon, 2014; Saarikallio & Erkkilä, 2007). The contents of HUMS Healthy – distractive relaxation, mood improvement, fostering of social connections – may indeed serve as relevant components of this endeavor. This is congruent with findings showing that music use reduces self-perceived stress particularly if it induces positive emotion (Helsing, 2012). In conclusion, HUMS Healthy is not a measure of adolescent wellbeing per se, but might be useful for investigating music use as a buffer against the impact of stressors and other ‘risky’ elements of adolescent life.

The HUMS was specifically developed to address musical engagement as an indicator of proneness for depression in youth. It may not be directly generalizable to other behavioral and mental health problems or age groups, and these connections may be worth future investigation. HUMS Unhealthy is more straightforward in its connection to depression than HUMS Healthy, and we therefore recommend HUMS Unhealthy to be considered the primary scale for measuring the proneness for depression. HUMS Healthy is, nevertheless, an important addition to the measure, as both our previous qualitative study (McFerran & Saarikallio, 2013) and the pilot phase comments clearly demonstrated that adolescents were reticent to engage with the idea that music could be unhealthy. Therefore, inclusion of only ‘negative’ items might elicit defensive reactions in respondents. Finally, due to this positive attitude of young people toward their music use (McFerran et al., 2013), the healthy items are prone to show a negative and unhealthy items a positive skew. The two items with highest skew (Music makes me feel bad about who I am/Music leads me to do things I shouldn't do) also had the lowest factor loadings and were among the ones that the adolescents most disliked during the pilot phase. These limitations are relatively minor, and the results demonstrate HUMS as a promising, pioneering instrument for assessing the health-relevance of adolescents’ music use.

In conclusion, HUMS is a comprehensive and valid instrument, for which the following forms of application are suggested. HUMS Unhealthy in particular is applicable as a screening instrument for identifying adolescents’ risky patterns of music engagement regarding proneness for depression. HUMS Healthy may be particularly applicable when measuring health-outcomes and studying musical engagement as a buffer/resource/protective feature against stressors and other risk factors. Both subscales can also be used as outcome measures for therapy interventions using music that deal with the treatment of depression and/or developing healthy relationships with music. Exact cutoff scores for different purposes are not presented, as it is important to retain a level of cautiousness in using HUMS as a diagnostic tool. This is because, while the correlation of HUMS unhealthy to depression is strong, it is still just .70. It should therefore be concluded that HUMS is not a direct measure of depression but an instrument to detect a risk for depression and other mental health problems in a nonintrusive way. Instead, it can be recommended that a high HUMS Unhealthy score would be followed up with a screening measure for depression and suicide risk. As a 13-item scale HUMS is compact and easy to administer, and the items contain language used and understood by English-speaking youth. It is theoretically solid and intelligible, and its reliability and validity are promising. It is likely to be of great value for both researchers and practitioners in the field of music and adolescent mental health.

## Supporting Information

**Appendix S1.** Healthy-Unhealthy Uses of Music Scale (HUMS).
