# Supplementary material for: Development and validation of the Healthy-Unhealthy Music Scale
Source: Child Adolesc Ment Health. 2015 May 18;20(4):210–7. doi: 10.1111/camh.12109 (PMC4690158; doi:10.1111/camh.12109)
Supplement: Supplementary file 1 — Appendix S1. Healthy-Unhealthy Uses of Music Scale (HUMS). [file camh0020-0210-sd1.pdf]

Healthy-Unhealthy Music Scale (HUMS)

Most people believe that music is a helpful part of their lives, but sometimes it's not. When you answer the questions below, please try to recall actual moments when music has been helpful and when it has not.

Please read each statement and mark how much it applies to you. Mark only one answer for each question.

		Never	Rarely	Some-times	Often	Always
1.	When I listen to music I get stuck in bad memories	☐	☐	☐	☐	☐
2.	I hide in my music because nobody understands me, and it blocks people out	☐	☐	☐	☐	☐
3.	Music helps me to relax	☐	☐	☐	☐	☐
4.	When I try to use music to feel better I actually end up feeling worse	☐	☐	☐	☐	☐
5.	I feel happier after playing or listening to music	☐	☐	☐	☐	☐
6.	Music gives me the energy to get going	☐	☐	☐	☐	☐
7.	I like to listen to songs over and over even though it makes me feel worse	☐	☐	☐	☐	☐
8.	Music makes me feel bad about who I am	☐	☐	☐	☐	☐
9.	Music helps me to connect with other people who are like me	☐	☐	☐	☐	☐
10.	Music gives me an excuse not to face up to the real world	☐	☐	☐	☐	☐
11.	It can be hard to stop listening to music that connects me to bad memories	☐	☐	☐	☐	☐
12.	Music leads me to do things I shouldn't do	☐	☐	☐	☐	☐
13.	When I'm feeling tense or tired in my body music helps me to relax	☐	☐	☐	☐	☐

Scoring instruction: HUMS Healthy: 3, 5, 6, 9, 13; HUMS Unhealthy: 1, 2, 4, 7, 8, 10, 11, 12. Answers are scored on a scale from 1 (never) to 5 (always).
